# Genome scans reveal signals of selection associated with pollution in fish populations of *Basilichthys microlepidotus,* an endemic species of Chile

**DOI:** 10.1038/s41598-024-66121-x

**Published:** 2024-07-08

**Authors:** Caren Vega-Retter, Noemi Rojas-Hernández, Jorge Cortés-Miranda, David Véliz, Ciro Rico

**Affiliations:** 1 Departamento de Ciencias Ecológicas, Facultad de Ciencias, Universidad de Chile, Las Palmeras #3425, Ñuñoa, Santiago Chile; 2 Centro de Ecología y Manejo Sustentable de Islas Oceánicas (ESMOI), Coquimbo, Chile; 3 Instituto de Ciencias Marinas de Andalucía (ICMAN), CSIC. Campus Universitario Río San Pedro, C. Republica Saharaui, 4, 11519 Puerto Real, Cádiz Spain

**Keywords:** Conservation biology, Environmental impact

## Abstract

The Maipo River catchment is one of Chile’s most polluted basins. In recent decades, discharges of untreated sewage and organic matter have caused eutrophication and water quality degradation. We employed the indigenous silverfish species *Basilichthys microlepidotus* as a model organism to investigate the process of adaptation and selection on genes influenced by pollution. Using variation at single nucleotide polymorphisms (SNPs), we determined the temporal stability of the population structure patterns previously identified in this species by varying SNPs. We also examined local adaptation to pollution-selected genes. Using the genotypes of 7684 loci in 180 individuals, we identified 429 and 700 loci that may be undergoing selection. We detected these loci using the FSTHET and ARLEQUIN outlier detection software, respectively. Both software packages simultaneously identified a total of 250 loci. *B. microlepidotus*' population structure did not change over time at contaminated or unpolluted sites. In addition, our analysis found: (i) selection of genes associated with pollution, consistent with observations in other organisms; (ii) identification of candidate genes that are functionally linked to the same biological processes, molecular functions and/or cellular components that previously showed differential expression in the same populations; and (iii) a candidate gene with differential expression and a non-synonymous substitution.

## Introduction

Biodiversity loss is a global issue due to anthropogenic influences like habitat fragmentation, degradation, climate change, and other stressors^1,2^. Mitigating biodiversity decline is a fundamental concern, with pollution being a significant factor in ecological and evolutionary change^3^. Human activities, such as agriculture, industrial, and residential, emit pollutants into soil and drainage systems, which infiltrate freshwater systems, causing contamination^4,5^. These substances can cause toxicity, affecting organisms, population dynamics, and ecosystem productivity^6^. However, most studies focus on individual responses^7^, leaving a limited understanding of population-level responses to pollution^8^. This knowledge is crucial for developing effective conservation strategies and policies, as it helps predict species' adaptation and survival in polluted environments. More research is needed to investigate the interactions among individuals within a population and their impact on a species' overall adaptive capacity. Recent studies have shown that certain genes in fish alter their expression profiles in response to pollution, such as CYP1A expression, which can lead to tumour formation^9^. Local adaptation, which results in genetic modifications at the genome level, can also contribute to an organism's ability to survive. For example, Whitehead et al.^10^ suggested that the contrasting survival rates of *Fundulus heteroclitus* in two populations in polluted regions may be due to genetic adaptation rather than flexible physiological responses implying that the evolution of pollution tolerance, which is specific to local environments, entails intricate patterns of gene expression and variation in genome sequences. Furthermore, despite genetic distances and exposure to diverse chemical combinations, Reid et al.^11^ found that wild killifish populations frequently targeted genes in the aryl hydrocarbon receptor pathway for selection. Yin et al.^12^ found that the pesticide 3-trifluoromethyl-4-nitrophenol (TFM) changed the expression patterns of hundreds of genes. In the sea lamprey Petromyzon marinus that was exposed to TFM, one allele showed higher expression levels. Identifying genes that undergo selection is the first step in understanding the impact of adaptation on biological responses to pollution and population survival.

Chile's Maipo River, a heavily polluted catchment area, supplies 70% of the region's drinking water and irrigation needs. The river basin, spanning 15,304 km^2^, is primarily made up of watercourses drained from snowmelt from the Andes Mountain Range^13,14^. With 7.11 million human residents, it accounts for 40% of the Chilean population, living in 163 regions, including Santiago^15^. Untreated sewage in the basin contributes to eutrophication and poor water quality^16^. The first wastewater treatment system only processes 25% of dirty water. A 2012 collection system was implemented to improve water quality^17^. However, contaminants like chemicals and heavy metals persist after treatment^18^. The basin's industrial concentration and mining activity contribute to heavy metal pollution^14^.

**Figure 1:**
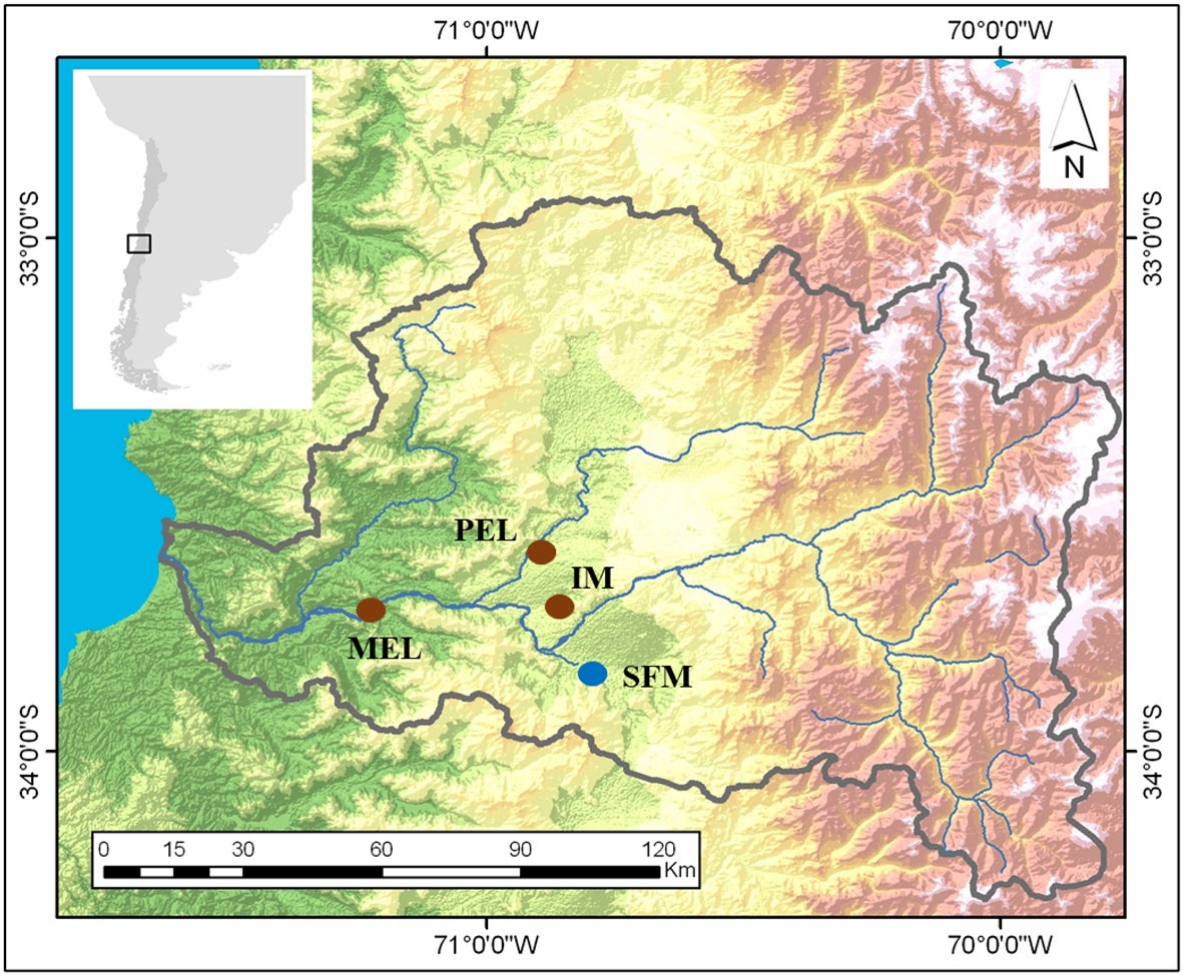
*B. microlepidotus* sampling sites in the Maipo River basin. Brown circles represent polluted sites, and the blue circle represents the non-polluted site. *SFM* San Francisco de Mostazal, *IM* Isla de Maipo, *MEL* Melipilla, *PEL* Pelvin. The map was modified from Veliz et al.^17^ using ArcGIS online (https://www.arcgis.com/index.html). Veliz et al.^17^ is published under the Creative Commons License CC BY 4.0.

Analyses of ten physicochemical parameters measured in 2007, 2011, and 2016 showed that some sites of the basin, such as Pelvin (PEL) and Melipilla (MEL), suffered chronic pollution, while others, such as San Francisco Mostazal (SFM), showed a consistently lower grade of pollution over time (Fig. 1)^13^. Other sites, like Isla de Maipo (IM), have suffered considerable water quality deterioration in recent years. Using 36 environmental variables (22 from water and 14 from sediments) measured in 2016, the same study found a clear separation between SFM and the other sites, PEL, MEL and IM^13^. Recent studies have demonstrated the impact of this pollution on the biota, specifically on the endemic silverside fish *Basilichthys microlepidotus* a vulnerable insectivorous atherinid that inhabits lakes and rivers from 28° to 39°S^19^. Vega-Retter et al.^20^ found the first evidence of selection as a response to pollution in silversides using AFLP. Later, RNA-Seq analysis on liver samples collected in 2012 revealed differences in gene expression among individuals inhabiting polluted and unpolluted sites. The samples from the population inhabiting MEL showed an upregulation of genes related to tumour suppression and progression, cell proliferation, and microtubules, while individuals from PEL showed an upregulation of genes related to apoptotic processes, among others^21^. The same study reported a heterozygote deficit and increase in the corresponding homozygote genotype frequency at both polluted sites (PEL and MEL) in an overexpressed gene related to tumour promotion and progression, suggesting possible selection for this gene^21^.

Evidence suggests that pollution-induced selection occurs in populations of *B. microlepidotus* residing in this basin. Despite this fish's presence in this basin, research on the genome-wide diversity patterns associated with its function is lacking. Therefore, the primary objectives of this study were: (i) to determine whether the population structure in the four basin areas (i.e. SFM, PEL, MEL, IM)^22^ remained unchanged over time; (ii) to identify genes impacted by pollution and closely examine the known functions of these potentially adaptive loci; (iii) to establish a connection between the functions of pollution-selected candidate genes and those that exhibited varying expression levels in previous studies at the same sites and species, and to determine if the nucleotide substitutions of genes exhibiting differential expression were synonymous or non-synonymous changes. To perform this study, we used a genotyping-by-sequencing approach and variation at single nucleotide polymorphisms (SNP).

## Results

A data set consisting of 19,205 single nucleotide polymorphisms (SNPs) was obtained from a cohort of 184 genotyped individuals. We successfully obtained a comprehensive set of 7684 loci and 180 individuals after applying quality filtering procedures (Supplementary Table S1). We observed no cases of individuals displaying genetic relatedness. The FSTHET software identified 429 loci as outliers, while the ARLEQUIN software recognised 700 loci as outliers. In addition, there were 250 loci that were identified as outliers by both programmes. Subsequently, the loci identified as outliers by FSTHET and ARLEQUIN, totalling 879 loci, were discarded to create the neutral SNP dataset. No deviations from Hardy–Weinberg Equilibrium (HWE) were detected in the data. However, a total of 94 pairs of SNPs exhibited a significant level of linkage disequilibrium (LD), so one SNP from each pair was excluded from the analysis. This resulted in a final dataset consisting of 6711 neutral SNP loci from 180 individuals. We then used this dataset to assess the genetic structure and gene flow between populations.

### Population genetic structure, genetic diversity, and contemporary gene flow

**Figure 2:**
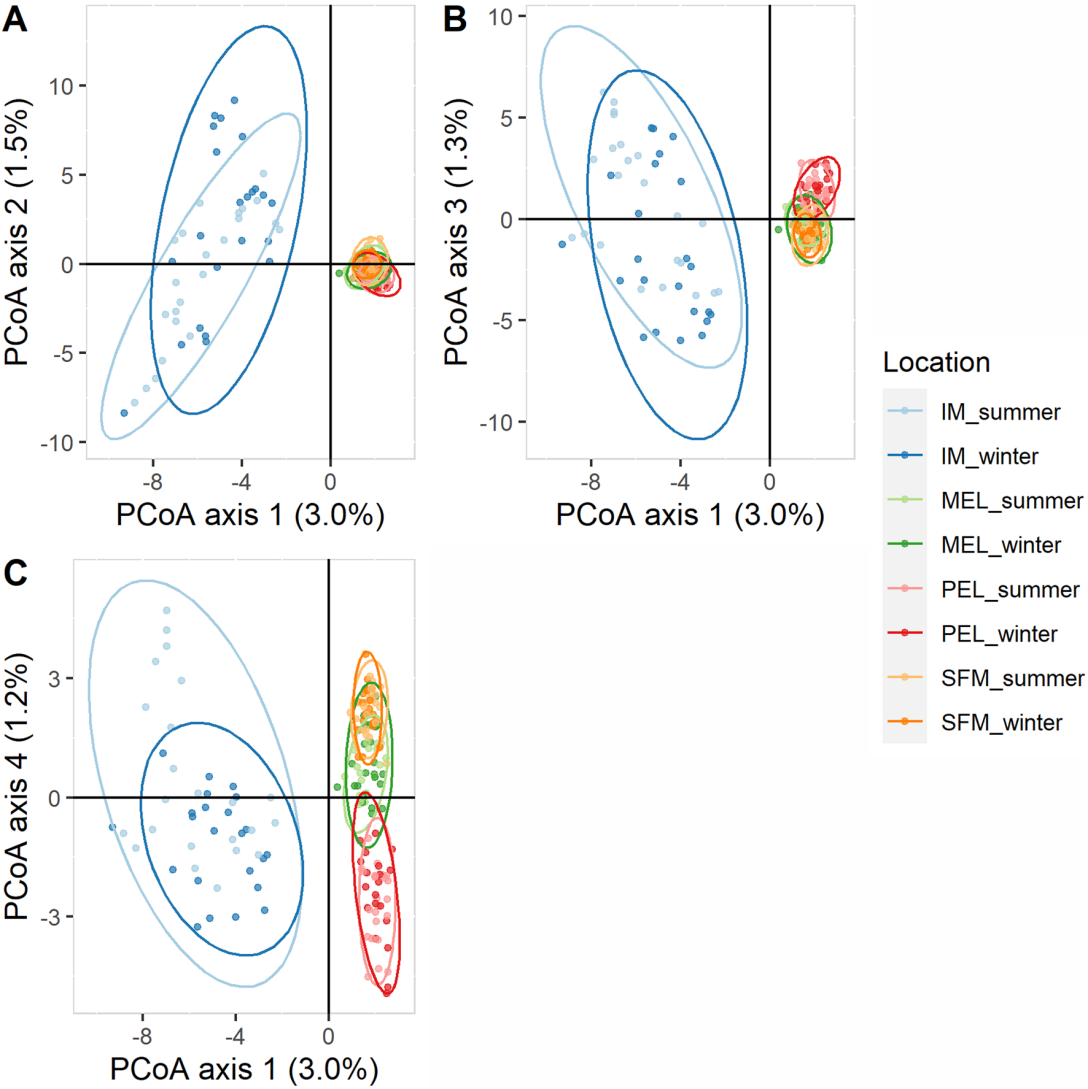
Principal coordinate analysis (PCoA) performed for *B. microlepidotus*. (**A**) The first and second principal components (x-axis and y-axis, respectively) capture 3% and 1.5% of the total variance, respectively. (**B**) The first and third principal components; the third principal components capture 1.3% of the total variance. (**C**) The first and fourth principal components; the fourth principal component captures 1.2% of the total variance. *PEL* pelvin, *MEL* melipilla, *IM* Isla de Maipo, *SFM* San Francisco de Mostazal.

**Figure 3:**
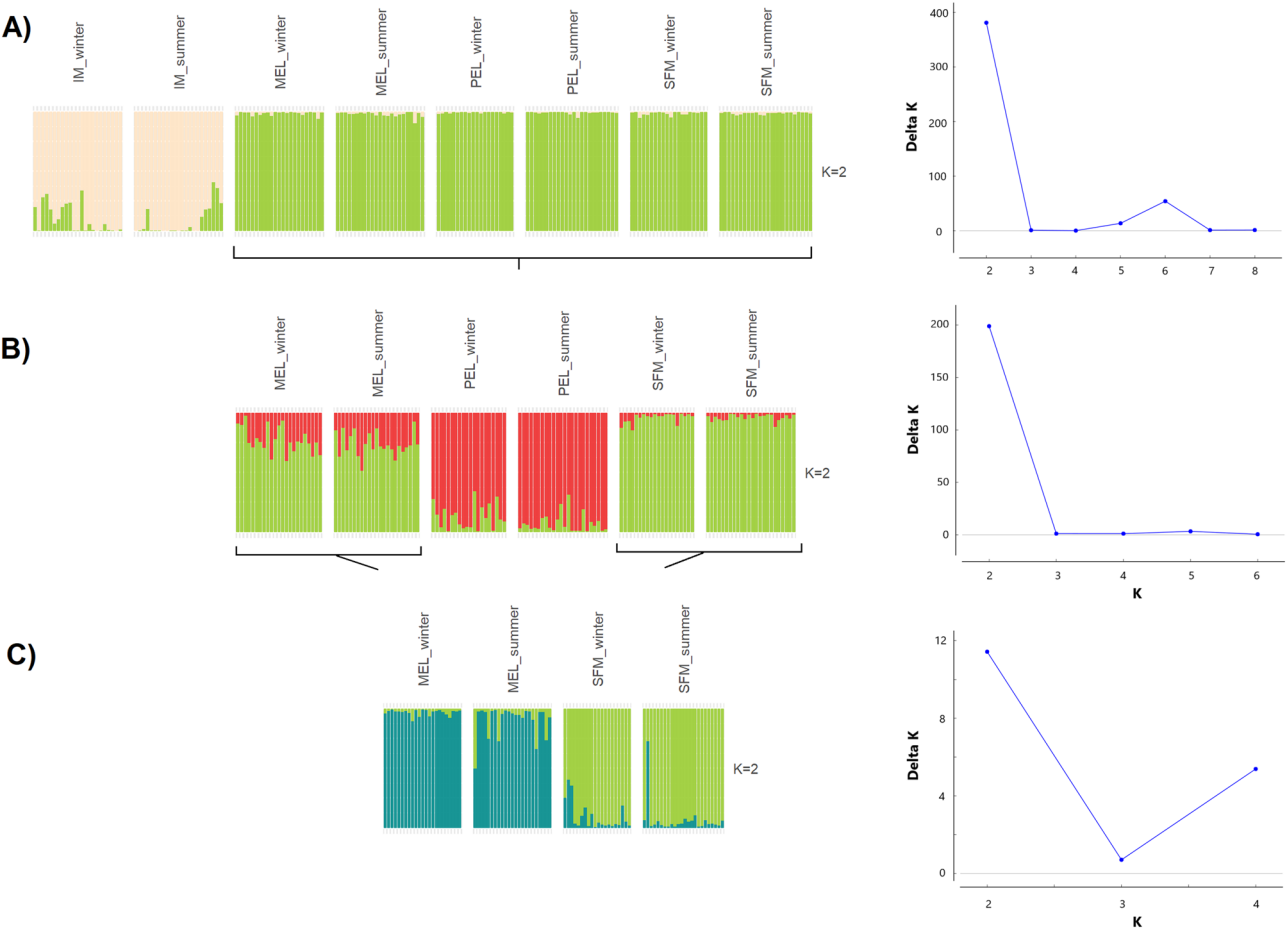
Structure plots for: (**A**) all the sites and seasons sampled showing two clusters, (**B**) without the IM site and its seasons showing two clusters, and (**C**) including only MEL and SFM sites, with the sampled seasons showing two clusters. In the right of each panel, the ΔK estimated using the method of Evanno et al.^59^ is displayed. *PEL* Pelvin, *MEL* Melipilla, *IM* Isla de Maipo, *SFM* San Francisco de Mostazal.

The F_ST_ analysis showed genetic diversity between all populations, with no statistically significant differences observed between seasons within sites. All pairwise comparisons between sites showed significant differences from zero. Notably, when comparing IM to all other sites, F_ST_ values were significantly greater than most other pairwise comparisons (Table 1). The principal coordinates analysis (PCoA) results show that the IM population is clearly separated from the other populations. This is clear from the way the data is organised along the first and second axes (Fig. 2A). In addition, the third and fourth axes show segregation of the SFM, MEL, and PEL populations (Fig. 2B,C). The analysis conducted revealed no discernible variation across seasons for each individual site, as can be seen in Fig. 2A–C. The initial analysis of STRUCTURE showed the existence of two distinct clusters, namely IM and SFM-MEL-PEL (Fig. 3A). However, after applying Evanno's approach to the second STRUCTURE analysis, which included only SFM-MEL-PEL and excluded IM, PEL was found to be separated from SFM-MEL (Fig. 3B). Using SFM and MEL, the analysis actually revealed two distinct populations. This is visualised in Fig. 3C, where the Evanno technique was used. It is noteworthy that the three STRUCTURE analyses revealed no significant differences between seasons within each site, as can be seen in Fig. 3A–C. Thus, the three methods showed variation between the sites but no variation between seasons.

**Table 1:** Pairwise F_ST_ and associated p-values for each sampling site and season were obtained after 1000 permutations.

	IM_win	MEL_sum	MEL_win	PEL_sum	PEL_win	SFM_sum	SFM_win
IM_sum	0.008	0.037*	0.038*	0.044*	0.043*	0.04*	0.04*
IM_win		0.032*	0.034*	0.038*	0.039*	0.034*	0.035*
MEL_sum			0.001	0.008*	0.007*	0.006*	0.005*
MEL_win				0.01*	0.01*	0.007*	0.006*
PEL_sum					0.0005	0.013*	0.013*
PEL_win						0.012*	0.012*
SFM_sum							0.0006

*SFM* San Francisco de Mostazal, *IM* Isla de Maipo, *MEL* melipilla, *PEL* pelvin, *Sum* summer, *win* winter.

*Denote significant p-values (p < 0.002).

The IM site had the lowest AR, H_O_, and H_E_ values, followed by PEL and SFM, with the MEL site showing the highest genetic diversity (Table 2). The current migration rate showed high values of self-recruitment for all populations (> 90%), with the exception of MEL (migration rate = 0.715), which showed high immigration values from the populations PEL and SFM (Table 3).

**Table 2:** Summary of genetic variables estimated for each population.

Population	AR	HO	HE	FIS
IM	1.71	0.179	0.179	0.014
MEL	1.90	0.195	0.201	0.040
PEL	1.87	0.189	0.196	0.046
SFM	1.89	0.193	0.200	0.046

*AR* allelic richness, *HO* observed heterozygosity, *HE* expected heterozygosity, *FIS* inbreeding coefficient, *SFM* San Francisco de Mostazal, *IM* Isla de Maipo, *MEL* melipilla, *PEL* pelvin.

**Table 3:** Current migration rate estimated for populations of *B. microlepidotus* in the Maipo River basin.

From/to	IM	MEL	PEL	SFM
IM	**0.98 (3.1E-04)**	0.007 (4.5E-05)	0.007 (5.4E-05)	0.007 (5.4E-05)
MEL	0.007 (3E-04)	**0.715 (0.042)**	0.013 (0,002)	0.041 (0.036)
PEL	0.007 (5.5E-05)	0.146 (0.056)	**0.972 (1.5E-04)**	0.008 (0.001)
SFM	0.007 (3.1E-04)	0.132 (0.087)	0.008 (0.002)	**0.945 (0.036)**

Each value represents a mean of five different runs. Standard deviations are shown in parentheses. Values in bold represent self-recruitment rates.

### Detection candidate loci under selection, function, and synonymous or non-synonymous substitution

**Figure 4:**
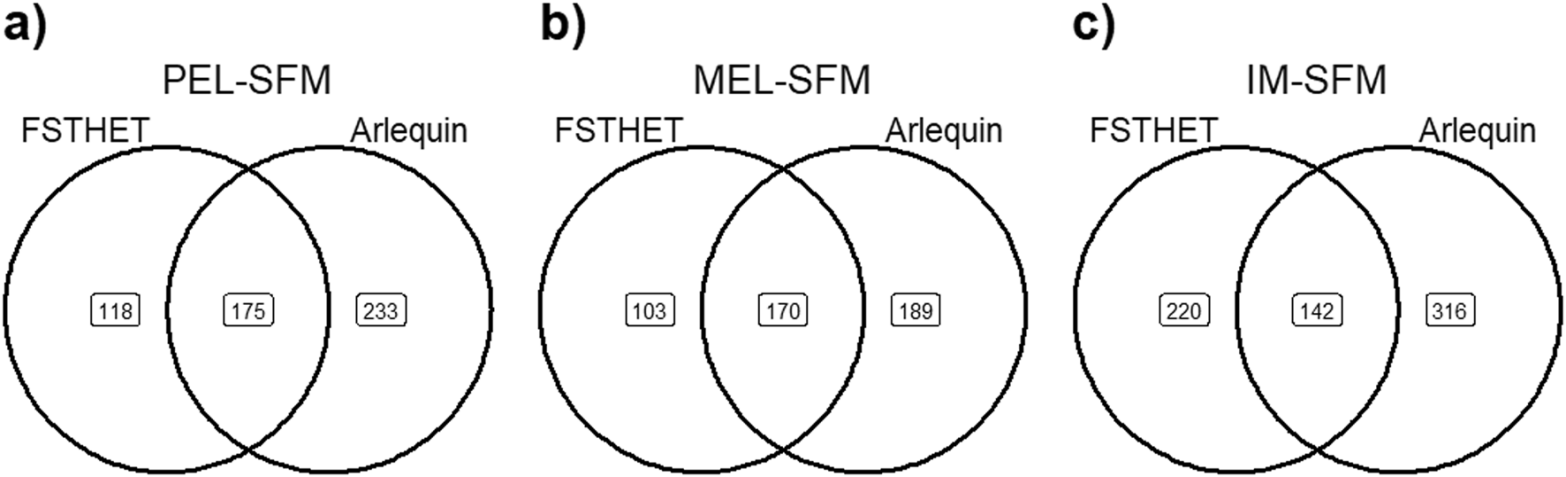
Venn diagram of the number of loci showing signatures of selection by each program, FSTHET and ARLEQUIN and loci detected by both programs for comparisons: (**a**) PEL-SFM, (**b**) MEL-SFM, and (**c**) IM-SFM.

**Figure 5:**
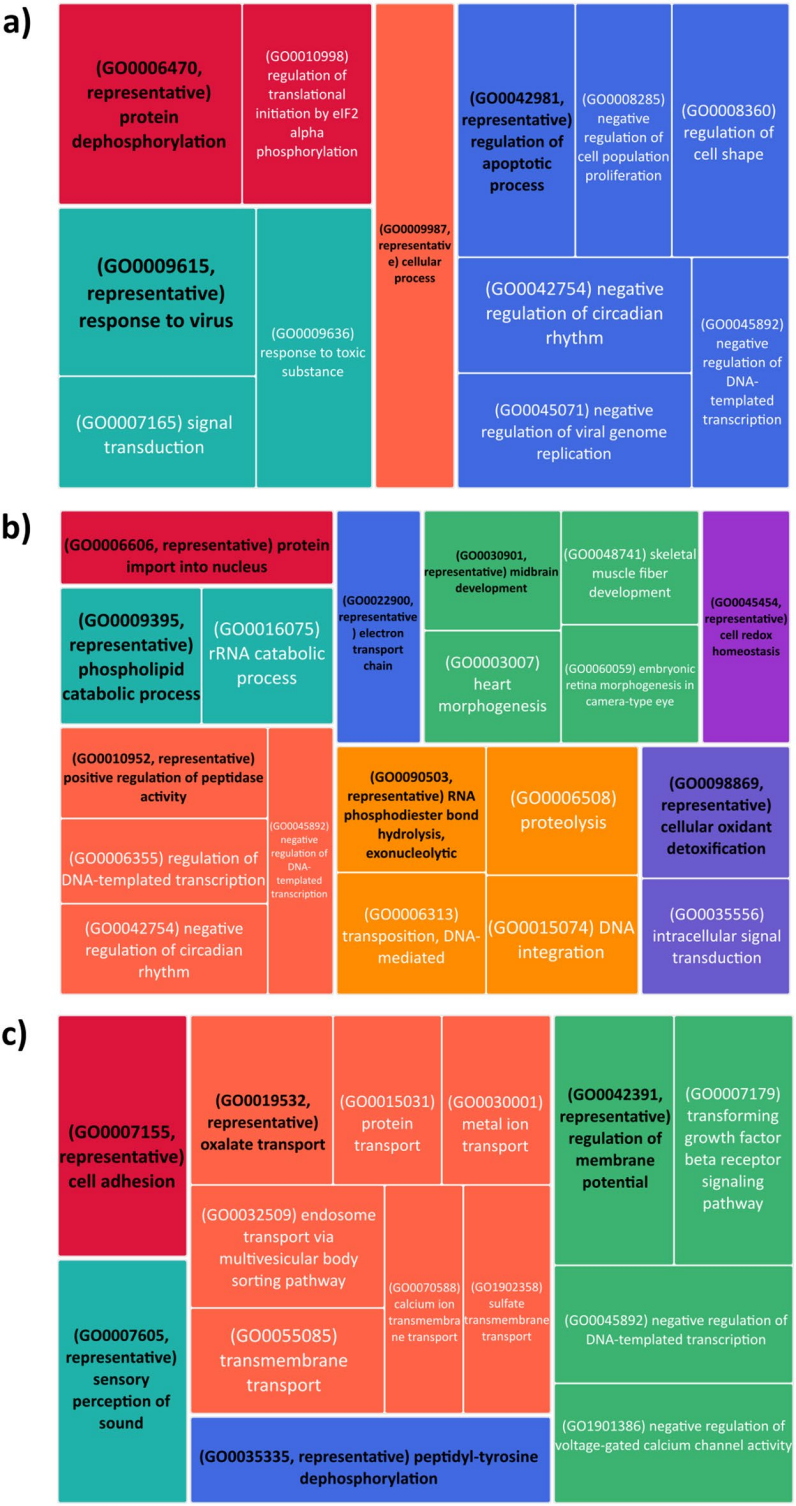
Tree map showing the biological processes involving the annotated genes detected as candidates to be under selection due to pollution across comparisons: (**a**) PEL-SFM, (**b**) MEL-SFM, and (**c**) IM-SFM. GO terms in black indicate the representative term: terms that are classified as similar after the semantic reduction process. *PEL* Pelvin, *MEL* Melipilla, *IM* Isla de Maipo, *SFM* San Francisco de Mostazal.

The outliers identified by the FSTHET method in the three comparisons ranged from 273 to 362 loci, but the outliers detected by the ARLEQUIN method ranged from 359 to 458 loci. The number of loci with selection signals identified by both programmes varied in different comparisons. The PEL-SFM comparison identified 175 loci, corresponding to 2.33% of the total number of loci analysed (Fig. 4a), while the IM-SFM comparison identified 142 loci, corresponding to 1.9% of the total number of loci analysed (Fig. 4c). Of the analysed loci, a range of 27 to 36 was subjected to blasting, while a range of 13 to 18 was subsequently annotated, as indicated in Supplementary Table S2. REVIGO analysis revealed that the gene candidates selected in the PEL-SFM comparison (Fig. 5a) were associated with Gene Ontology (GO) terms related to biological processes. These GO terms include response to viruses and dangerous compounds, as well as regulation of the apoptotic process. The comparison between MEL and SFM showed that the genes that showed signs of selection correlated with GO keywords such as cell redox homeostasis, cellular oxidant detoxification, proteolysis, and electron transport chains, among others (Fig. 5b). When we looked at the IM and SFM comparison, we saw biological processes associated with oxalate transport and membrane potential control, among other activities (Fig. 5c). In this same comparison, genes related to molecular functions involved in the binding of metal ions and the transmembrane transport of metal ions were identified, as well as a GO term associated with the transmembrane transport of oxalate (GO0019531: oxalate transmembrane transporter activity). The genes that showed evidence of selection in the MEL-SFM comparison had molecular function GO keywords connected with the cytoskeleton, such as motor activity in the cytoskeleton and actin binding (see Supplementary Fig. S1). The MEL site showed the presence of a cellular component GO term associated with the cytoskeleton, namely the myosin complex. Conversely, the IM and PEL sites had membrane-associated cellular components, as shown in Supplementary Fig. S2.

**Figure 6:**
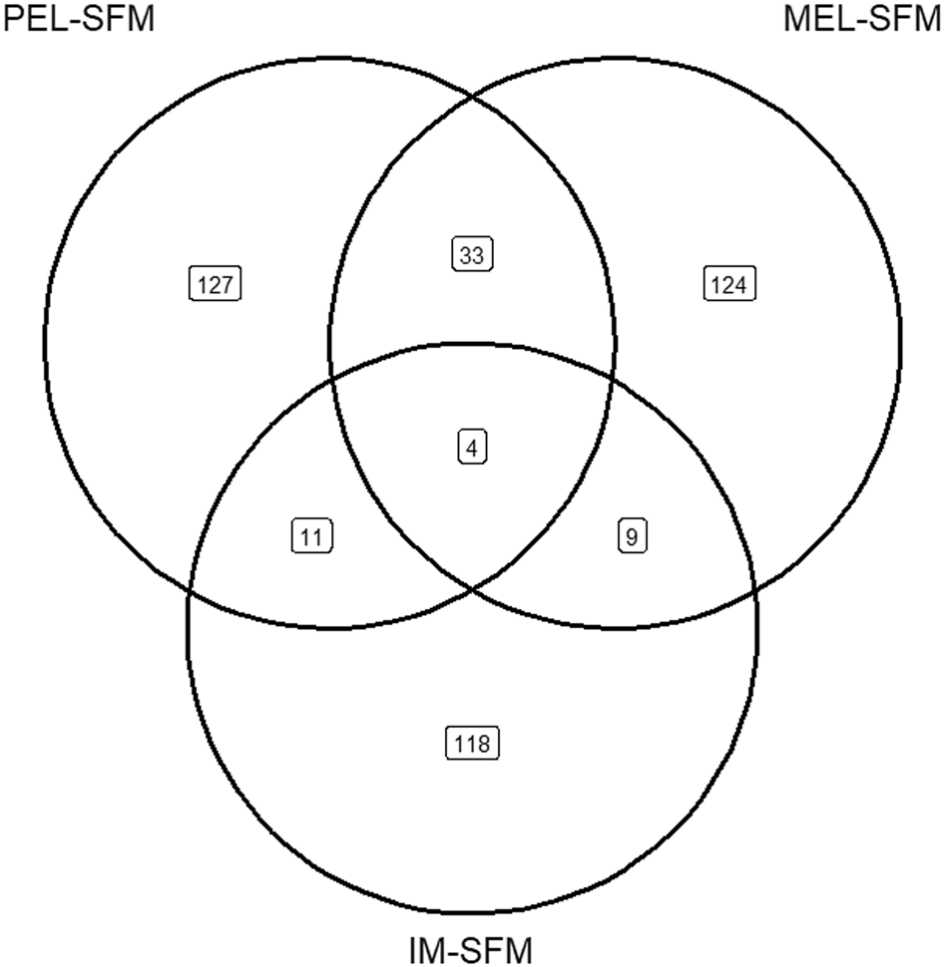
Venn diagram showing the shared outliers loci detected for each comparison.

The comparison between SFM-PEL and SFM-MEL revealed the largest number of common loci, totalling 33, which were identified as potential candidates for selection due to contamination. Notably, both the SFM-IM and SFM-PEL comparisons identified a total of 11 loci as potential candidates for selection, while the SFM-IM and SFM-MEL comparisons identified 9 loci as common. Finally, four genes showed signs of selection across all three sites, namely PEL, MEL, and IM (Fig. 6). Of the loci that were shared, only two were annotated. The first locus was located at the IM and MEL sites, which is associated with the cellular component membrane. The second locus is located in the PEL and MEL sites and is involved in the biological processes of negative regulation of circadian rhythm and negative regulation of DNA-driven transcription. In 2018–2019, we identified two loci in our dataset that deviated significantly from the expected patterns and that, interestingly, also showed differential expression in the RNA-Seq study from 2016 (RNA-Seq data available in Cortés-Miranda et al.^13^). We found a correlation between one of the genetic markers and individuals from the MEL locus, while the other marker was associated with the PEL locus. The locus in MEL could not be fully described, but the locus in PEL (locus identity 38199422-57) corresponds to a protein that has not yet been fully characterised. This protein had a non-synonymous substitution at position 427 within codons 427–429 of the coding sequence (AAT/GAT). In individuals originating from the non-polluted SFM region, both alleles occurred at comparable frequencies (A = 54.55%; G = 45.45%). In contrast, individuals from the polluted site PEL mostly possessed an adenine at the same position (79.55%). The adenine base is responsible for the coding of the amino acid asparagine, which is classified as a neutral polar amino acid. Guanine, on the other hand, codes for the amino acid aspartic acid, which is characterised as a negatively charged amino acid.

## Discussion

The main objective of this work was to identify potential genes under selection pressure due to pollution in wild populations of *B. microlepidotus* throughout the Maipo River Basin. First, we assessed the temporal stability of the four natural populations used in the present study. The analysis demonstrated the temporal stability of the four examined populations. This conclusion was drawn based on the fact that these populations were sampled in 2011 and 2012, as reported by Vega-Retter et al.^22^. Furthermore, no recognisable fluctuations between seasons were observed in this study. The site designated as MEL showed the most remarkable results in terms of diversity indices, possibly due to the considerable influx of immigrants, mainly from the PEL and SFM sites. Previous studies of the same populations using microsatellite markers have consistently shown a similar trend: a relatively low rate of self-recruitment in the MEL population, which is the lowest of all populations, with immigration coming mainly from the PEL and SFM populations^22,23^. It is noteworthy that, despite the considerable migration from PEL and from SFM in MEL, no recognisable genetic homogeneity could be detected. One possible explanation for this phenomenon is the occurrence of a potential local adaptation process that could be due to spatially divergent selection. This concept is consistent with the patterns of local adaptation through spatially varying selection discussed by Savolainen et al.^24^, which were found in a number of different species.

Pollution-induced selection shared some genes and processes, but most of them are specific to a single population. This is possibly due to the characteristics of the pollutants and their combinations being different at each site, suggesting that different genes are involved in the process of adapting to a particular pollutant or combination of them. The results of our study showed that a range of 1.9 to 2.33% of the loci analysed showed detectable evidence of selection related to pollution. The individuals living at Pelvin showed evidence of selection based on biological processes related to responses to viruses (Table S3). Several studies have shown that changes in the microbial community provide evidence of sewage discharges, urban runoff, and the discharge of water from animal farms in close proximity to the river. These inputs are associated with potential pathogens, as suggested by Suzzi et al.^25^ and Nagarajan et al.^26^. In a study conducted by Cohen^27^, it was shown that individuals of the species *Fundulus heterociclus* that were heavily parasitized at a site with high PCB contamination exhibited an increased occurrence of population-specific substitutions in the α-helix segment of the derived antigen binding region of the major histocompatibility complex (MHC). Samples from PEL showed selection signatures in genes associated with the regulation of apoptosis (Table S3). In a previous study by Vega-Retter et al.^21^, differential expression was observed at the same site for samples from 2012. This differential expression was characterised by an enrichment of molecular functions related to apoptosis, which is a cellular process highly regulated and controlled. It plays a central role in the establishment and maintenance of homeostasis in all metazoan organisms^28^. It can be affected by different categories of pollutants, including metals, persistent organic pollutants, and pesticides, as discussed in the study by AnvariFar et al.^29^. Therefore, the study shows that the apoptotic process is an important focal point for the potential effects of pollution-driven selection.

Our research at Melipilla revealed evidence of selection signalling in genes associated with cellular redox homeostasis, cellular detoxification of oxidants, proteolysis, and the electron transport chain, among others (Table S3). Steinhilber et al.^30^ have found evidence for the control of antioxidants by hormones, including melatonin, which has the ability to influence the expression of genes associated with antioxidant activity. Fish have evolved several mechanisms to mitigate the harmful effects of reactive oxygen species known to result from environmental pollution^31^. It is important to recognise the importance of maintaining cellular redox homeostasis as a fundamental mechanism for adaptation to a pollutant-laden environment^32,33^. We conducted the present study using the MEL database, which revealed significant evidence for selection on cytoskeleton-related GO terms, particularly cellular components and molecular functions. Vega-Retter et al.^21^ observed in their study an enrichment of biological processes, molecular functions, and cellular components related to the cytoskeleton, focussing in particular on microtubules, among the genes overexpressed at the same site. Several studies have documented changes in cytoskeleton-related functions, proteins, and gene expression in response to pollution in different animals and under different pollution situations^34–37^. Furthermore, proteomic analyses demonstrated that the cytoskeleton is one of the cellular targets frequently affected by stress^38^. According to Aseervatham^39^, cytoskeletal dysfunction is linked to the development of various diseases, including cancer. The identification of changes in gene expression associated with microtubules by Vega-Retter et al.^21^ together with the detection of selection signatures in genes related to the cytoskeleton, emphasises the importance of studying the effects of this association in order to understand the lasting effects of pollution on fish populations at this particular site.

There is evidence of selective pressure on genes at Isla Maipo that are involved in biological processes and molecular functions of oxalate transport (Table S3). Oxalic acid and oxalates are used in many household products, such as bleaches, metal cleaners, and paint removers. These compounds are also used in industry for engraving, lithographic processes, metallurgy, and medicine^40^. Previous research has shown that inadequate management of wastewater systems can lead to an increase in oxalate concentrations in sediments^41,42^. Urbanisation and industrial growth in Chile, especially in the immediate vicinity of Isla Maipo, could be associated with a deterioration in the quality of local water resources. This decline in water quality could possibly explain the observed selection of loci involved in the transport of this specific chemical component. Analysis of molecular functions using GO keywords revealed the presence of selection signatures in genes associated with metal ion binding and metal ion transmembrane transporter activity. This finding suggests that metal contamination may play an important role at this particular site. Water quality degradation increased recently at the IM site^13^ compared to the results of previous studies conducted in our laboratory^20–22^. The recent observed decline in water quality is consistent with the finding that this particular site had the lowest number of genes showing signs of selection pressure when compared with the sites with long term pollution, PEL and MEL.

Biological processes that show signs of selection in both PEL and MEL sites include the negative regulation of circadian rhythm and DNA-templated transcription (Table S3). Previous studies have documented the existence of interactions between chemical toxicity and the circadian rhythm, mostly attributed to the rhythmic activity of detoxification enzymes or to perturbations in the formation of rhythms by xenobiotics^43^. Both locations also showed a similar feature of negative control of DNA-driven transcription. The effects of various contaminants on the alteration of transcriptional regulation have been extensively documented in a variety of animals, including fish species^36,44–46^.

Based on the observed number of loci displaying indications of selection at various study sites, our analysis has demonstrated that a minimum of 18.3% (PEL), 21.2% (MEL), and 19% (IM) of these loci exhibited similarity to coding regions when subjected to BLAST searches utilising the contigs derived from the RNA-Seq experiments conducted in 2012 and 2016. However, there was no overlap between the genes showing selection signals and the genes showing differential gene expression identified in the 2012 RNA-Seq analysis^21^. Interestingly, two specific loci that showed signs of selection as a result of pollution in this study were also found to have differential expression in an RNA-Seq analysis performed in 2016^13^. A non-synonymous substitution in one of the identified genes, which corresponds to a non-characterised protein, results in the predominance of a neutral polar amino acid (asparagine) over a negatively charged molecule (aspartic acid) in this allele. This substitution was observed more frequently at the polluted site than at the reference site, where both alleles were present at a similar frequency. Crispo et al.^47^ described a non-synonymous variation in the MHC gene of *Rhinichthys cataractae*, a fish species living in three rivers in Alberta that has been impacted by municipal and agricultural activities. Based on the available evidence of selection, differential expression, and the occurrence of non-synonymous substitution, it is postulated that this particular gene may be involved in the process of adaptation to pollution. Therefore, it is of utmost importance to include this gene in future studies to assess its functionality and the possible consequences of the observed amino acid change.

In the context of field studies, such as the present study, the presence of confounding environmental factors can complicate the interpretation of adaptation to one or more stressors^48^. Our results indicated the presence of a stable population structure for *B. microlepidotus* at both contaminated and unpolluted sites^20–22^ and revealed: (i) evidence of selection on genes associated with pollution, which is consistent with observations in other organisms; (ii) identification of candidate genes that are functionally linked to the same biological processes, molecular functions, and/or cellular components that previously showed differential expression in the same populations; and (iii) identification of a candidate gene that shows differential expression and a non-synonymous substitution. Thus, our results suggest that there is a genetic basis for adaptation to pollution on the silverside through genes associated with similar biological processes, molecular activities, and cellular components that have different levels of expression. The results of this research will improve our understanding of the molecular processes and their interplay that determine the adaptive capacity of wild populations to complex environmental stressors and pollutants.

### Conservation implications

The IM population showed the most reduced genetic diversity and the highest rate of self-recruitment, with almost negligible migration exchange with the other populations in the river basin. Similar results were found in a previous study^22^, but there are no obvious geographical barriers that explain this fact. Further, the high genetic diversity detected at Melipilla could be explained by the high number of immigrants received from PEL and SFM sites. Thus, these results emphasise the need to protect: i) the IM population in order to improve its genetic diversity and enable its long-term survival; and ii) the migration of individuals from PEL and SFM to MEL to conserve its high genetic diversity.

## Materials and methods

### Sampling sites and sample collection

We conducted fieldwork in the Maipo River Basin in Chile during the winter of 2018 and the summer of 2019. We selected a total of four sites for sampling. One site, San Francisco de Mostazal (SFM, 33° 58′ 19.97″ S, 70° 42′ 56.49″ W), was previously identified as unpolluted in several studies^17,21, 22^. On the other hand, three other sites, namely, Melipilla (MEL, 33º 42′ 49,988″ S, 71º 12′ 39,13″ W) and Pelvin (PEL, 33º 36′ 21″ S, 70º 54′ 33″ W) were classified as polluted, while Isla de Maipo (IM, 33º 44′ 58″ S, 70º53′ 26″ W) has recently suffered a water quality degradation, being also considered as contaminated site in this study^13^ (Fig. 1). A total of 20–24 individuals of *B. microlepidotus* were collected by using a low impact electrofishing device that did not harm the fish at each site in each season, as indicated in Supplementary Table S1. We removed a small portion of the anal fin from each specimen and preserved it in 95% ethanol. The fish were then kept in a controlled environment with clean and oxygenated water for 20 min before being released back into the river. The protocols used in this study were approved by the Ethics Committee of the Universidad de Chile, complied with the laws in force in Chile (Resolución Exenta No. 3078 Subsecretaria de Pesca) and adhered to the ARRIVE guidelines. We performed all methods in accordance with the relevant guidelines and regulations.

### Sequencing and data filtering process

A small biopsy from each sample was sent to Diversity Arrays Technology Pty Ltd (DArT) in Canberra, Australia, for DNA extraction and sequencing using DArTSeq technology. Proprietary genotyping by sequencing DArTseq™ reduced-representation libraries were prepared as described by Kilian et al.^49^and Sansaloni et al.^50^. The DArTSeq library was sequenced using an Illumina Hiseq 2500 DNA Sequencing System. We performed data filtering using the dartR package^51,52^ in the R 4.3.0 environment^53^. In our study, we excluded SNP sequences from the dataset based on several criteria. First, we removed secondary SNPs, i.e. SNPs that occur in addition to the primary SNP of interest. Second, we excluded SNPs that were outside the trimmed sequence to ensure that only SNPs within the specified region were considered. Third, we excluded SNPs with an average read depth of less than 5 or more than 150, as these values were considered to be outside the desired range. We also excluded SNPs with a repeatability of less than 99% (repAvg), indicating a lack of consistency in the reads. We also excluded monomorphic loci, i.e. genetic loci with only one variant. We also removed loci with a locus call rate below 95%, indicating a low proportion of successful genotype calls. Finally, we did not include SNPs with a minor allele frequency (MAF) of less than 0.01, indicating a low frequency of the less common allele. In addition, an assessment of genomic relatedness was performed using the genomic relatedness matrix implemented in the dartR package. Finally, all individuals missing more than 5% of the data were excluded from the analysis.

### Population genetic structure, genetic diversity, and contemporary gene flow

To analyse the genetic structure of the population and assess its stability over time, we applied a method that involves the identification of outlier loci through a comprehensive comparison across all sites and seasons. This comparison was performed using smoothed quantiles of extreme F_ST_ values relative to their heterozygosity, implemented using the R package FSTHET^54^ with an alpha of 0.05. Additionally, coalescent simulations were performed using the software ARLEQUIN version 3.5.2.2v^55^, using the hierarchical island model (H), 20,000 simulations, 100 demes, 10 groups, and an alpha of 0.05. All loci that were recognised as outliers in both approaches were excluded to create a neutral SNP dataset. This dataset was then subjected to a test for deviation from Hardy–Weinberg equilibrium (HWE) using the false discovery rate (FDR) method implemented in the R package dartR. All single nucleotide polymorphisms (SNPs) that showed a significant deviation from Hardy–Weinberg equilibrium (p < 0.05) were excluded from the analysis. Finally, one of the pairs of single nucleotide polymorphisms (SNPs) that were found to have linkage disequilibrium (LD) was eliminated using PLINK2 software^56^ by using a squared allele number correlation (r2) criterion of 0.2.

The genetic structure of the population was assessed using neutral loci and three methods. First, estimation of genetic differences between pairs of sites was performed using F_ST_ statistics in the GENETIX programme as described by Belkhir^57^. Statistical significance was estimated using a total of 1000 permutations. To mitigate type I errors, a Bonferroni correction with a significance level of α = 0.002 was performed. A Principal Coordinates Analysis (PCoA) was performed to analyse the data and visually represent the genetic similarities between the populations. For this purpose, the function gl.pcoa from the dartR package^51^ was used. Finally, we used a Bayesian clustering and assignment method performed with the programme STRUCTURE 2.3.4^58^. The models for admixture and correlated allele frequencies were run with 300,000 iterations and a burn-in of 300,000 Markov Chain Monte Carlo (MCMC) iterations for each K. The procedure was repeated four times, from K = 1 to K = 9. To identify a possible population structure that is small but statistically significant, an additional analysis with identical parameters was performed for those groups that showed no differentiation in the first STRUCTURE study. The approach proposed by Evanno et al.^59^ was used in the STRUCTURE HARVESTER programme^60^ to determine the most likely value of K for our data set. The three approaches used in this study allowed for the identification of the genetic population structure. Subsequently, allelic richness (AR), observed heterozygosity (H_O_) and estimated heterozygosity (H_E_) were determined for each population using the dartR package^51^. The estimation of the inbreeding coefficient (F_IS_) for each population was performed with the GENETIX software using 5000 permutations.

To assess the current extent of gene flow, we used the BAYESASS3-SNPs (BA3-SNPS) software^61,62^. The study was conducted considering the genetic structure of the population previously established during a burn-in period of 2,000,000 iterations followed by 2,000,000 iterations after burn-in, with sampling every 100 iterations. The parameters for mixing, i.e. migration rates, allele frequencies, and inbreeding coefficients, were assigned values of 0.08, 0.25 and 0.02, respectively. To evaluate consistency, a total of five separate runs were performed, each starting with different seeds. The results were presented as the mean value of the migration rate from 5 different iterations.

### Detection of candidate loci under selection, function, and synonymous or non-synonymous substitution

Once the population's genetic structure was determined as described above, candidate loci that might be affected by pollution were identified using the original data set that was created after the filters were applied. We then compared the clean and polluted sites (SFM-PEL, SFM-MEL, and SFM-IM) using the R package FSTHET^54^ and the software ARLEQUIN^55^. We considered the SNPs detected by both programmes as candidate loci selected due to environmental contamination and discarded the SNPs detected by only one of the methods. To identify the function of the loci classified as candidates, we compared these sequences with our local database built with contigs from RNA-Seq assays performed in 2012^21^ and 2016^13^ for this species. We performed a second BLAST using the BLASTx function of the BLAST2GO software^63^ to map and annotate the candidate loci selected due to pollution. In order to reduce and summarise the GO term list obtained from the three independent comparisons, term clustering was performed using REVIGO^64^. REVIGO is based on semantic similarity measures; we used the default parameters (i.e. mean (0.7) cut-off value, removal of outdated GO terms, use of the Uniprot database and SimRel as semantic similarity measure).

As a complementary analysis, we identified the candidate loci under selection due to pollution detected in this study, which also showed differential expression in the RNA-Seq data performed in 2012 or 2016. For these genes, we determined whether the SNPs corresponded to synonymous or non-synonymous substitutions at the polluted site. We performed an NCBI BLASTx^65^ for this purpose, with the contig and/or the sequence obtained by DArT. Once we identified the sequence or contig, we used the CDS of the gene to ascertain the type of substitution.

### Supplementary Information

Supplementary Information.

## Data Availability

Data available at 10.34691/UCHILE/OLOEPP.
