# Supplementary material for: Genome scans reveal signals of selection associated with pollution in fish populations of Basilichthys microlepidotus, an endemic species of Chile
Source: Sci Rep. 2024 Jul 8;14:15727. doi: 10.1038/s41598-024-66121-x (PMC11231317; doi:10.1038/s41598-024-66121-x)
Supplement: Supplementary file 1 — Supplementary Information. [file 41598_2024_66121_MOESM1_ESM.docx]

Supplementary material

**Genome scans reveal signals of selection associated with pollution in fish populations of *Basilichthys microlepidotus,* an endemic species of Chile**

Caren Vega-Retter^1^, Noemi Rojas-Hernández^1^, Jorge Cortés-Miranda^1^, David Véliz^1,2^, Ciro Rico^3*^

(1) Universidad de Chile, Ciencias Ecológicas, Ciencias, Las Palmeras #3425, Ñuñoa, Santiago, Chile.

(2) Centro de Ecología y Manejo Sustentable (ESMOI), Coquimbo, Chile

(3) Instituto de Ciencias Marinas de Andalucía (ICMAN), Consejo Superior de Investigaciones Científicas (CSIC), Campus Universitario Río San Pedro, C. Republica Saharaui, 4, 11519 Puerto Real, Cádiz, España.

*Corresponding author: ciro.rico@csic.es

Table S1. Number of individuals sampled at each site and season and individual number of each site and season after the filtering process. SFM: San Francisco de Mostazal, IM: Isla de Maipo, MEL: Melipilla, PEL: Pelvin. sum: Summer, win: Winter.

|  | Initial number of individuals | Number of individuals after filter process |
| --- | --- | --- |
| SFM_sum | 24 | 24 |
| SFM_win | 20 | 20 |
| IM_sum | 23 | 23 |
| IM_win | 23 | 23 |
| MEL_sum | 23 | 23 |
| MEL_win | 23 | 23 |
| PEL_sum | 24 | 24 |
| PEL_win | 24 | 20 |
|  |  |  |

Table S2. Number of loci showing signatures of selection by each program, FSTHET and ARLEQUIN, loci detected by both programs, blasted and annotated for each comparison. PEL: Pelvin, MEL: Melipilla, IM: Isla de Maipo, SFM: San Francisco de Mostazal.

| Comparison | FSTHET | ARLEQUIN | Shared | Blasted | Annotated |
| --- | --- | --- | --- | --- | --- |
| PEL-SFM | 293 | 408 | 175 | 32 | 13 |
| MEL-SFM | 273 | 359 | 170 | 36 | 18 |
| IM-SFM | 362 | 458 | 142 | 27 | 14 |

Table S3. SNPs showing signs of selection, population/s in which were detected and its associated annotation, representative biological process and GO term.

| SNP | Population/s detected as outliers | Annotation | Representative biological process | Representative GO term |
| --- | --- | --- | --- | --- |
| 38195342-19-G/A | IM-MEL | VWFA and cache domain-containing protein 1 | Membrane | GO:0016021 |
| 100009582-26-A/G | MEL-PEL | CLOCK-interacting pacemaker | Negative regulation of circadian rhythm | GO:0042754 |
| 38194402-11-C/T | PEL | Interferon-induced, double-stranded RNA-activated protein kinase | Response to virus | GO:0009615 |
| 38194402-11-C/T | PEL | Interferon-induced, double-stranded RNA-activated protein kinase | Response to toxic substance | GO:0009636 |
| 38194402-11-C/T | PEL | Interferon-induced, double-stranded RNA-activated protein kinase | Regulation of apoptotic process | GO:0042981 |
| 38199557-31-T/C | PEL | Dual adapter for phosphotyrosine and 3-phosphotyrosine and 3-phosphoinositide | Protein dephosphorylation | GO:0006470 |
| 38200468-67-T/C | MEL | Thioredoxin reductase 1, cytoplasmic-like | Cell redox homeostasis | GO:0045454 |
| 38200468-67-T/C | MEL | Thioredoxin reductase 1, cytoplasmic-like | Cellular oxidant detoxification | GO:0098869 |
| 38206751-19-T/C | MEL | Transmembrane protease serine 9-like | Proteolysis | GO:0006508 |
| 38200468-67-T/C | MEL | Thioredoxin reductase 1, cytoplasmic-like | Electron transport chain | GO:0022900 |
| 38196651-68-A/C | IM | Prestin | Oxalate transport | GO:0019532 |


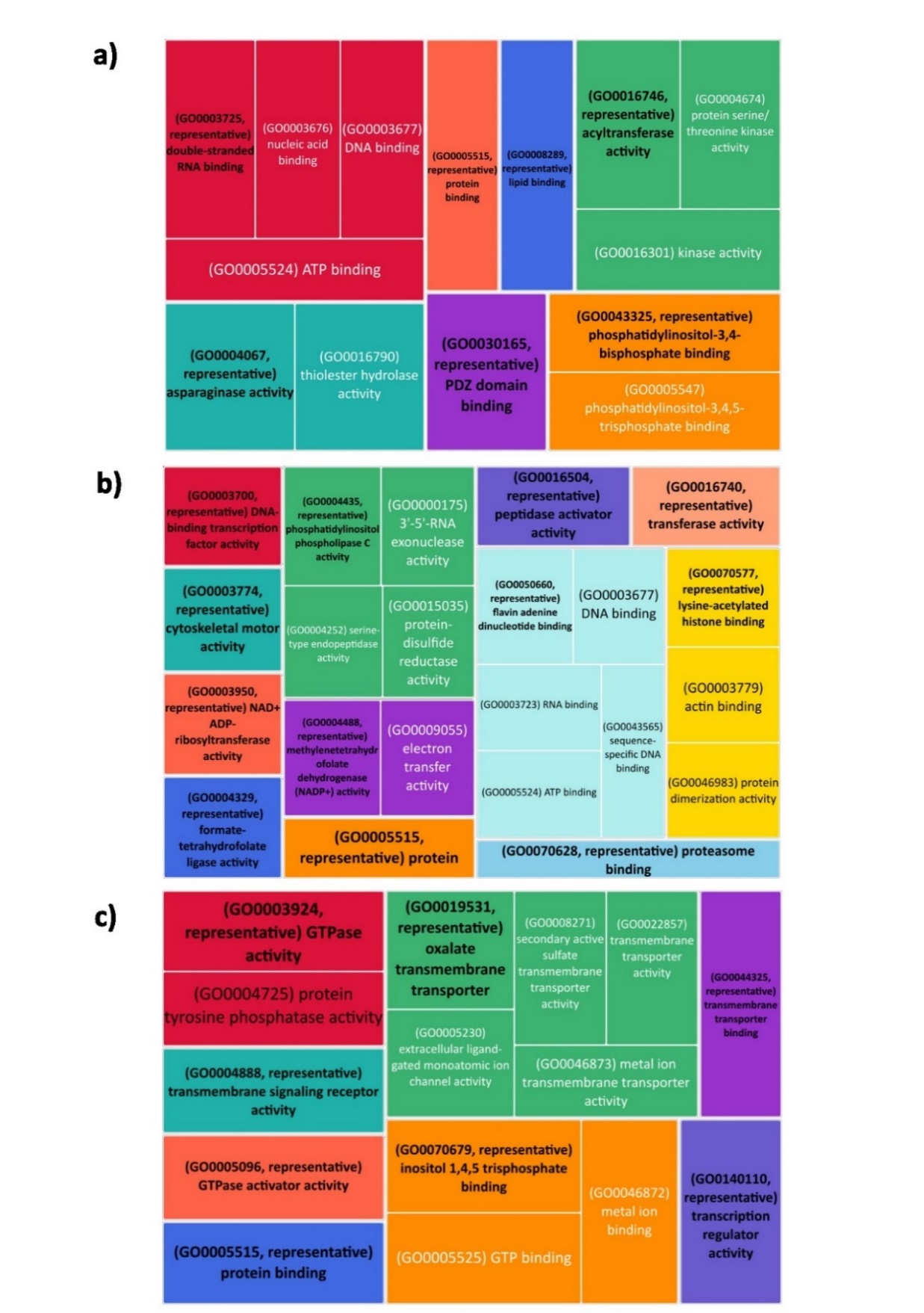


Figure S1. Treemap showing the molecular functions in which are involved the genes detected as candidate to be under selection due to the pollution in the comparison: a) PEL-SFM, b) MEL-SFM and, c) IM-SFM. GO terms in black indicate the representative term: terms that are classified as similar after the semantic reduction process. PEL: Pelvin, MEL: Melipilla, IM: Isla de Maipo, SFM: San Francisco de Mostazal.


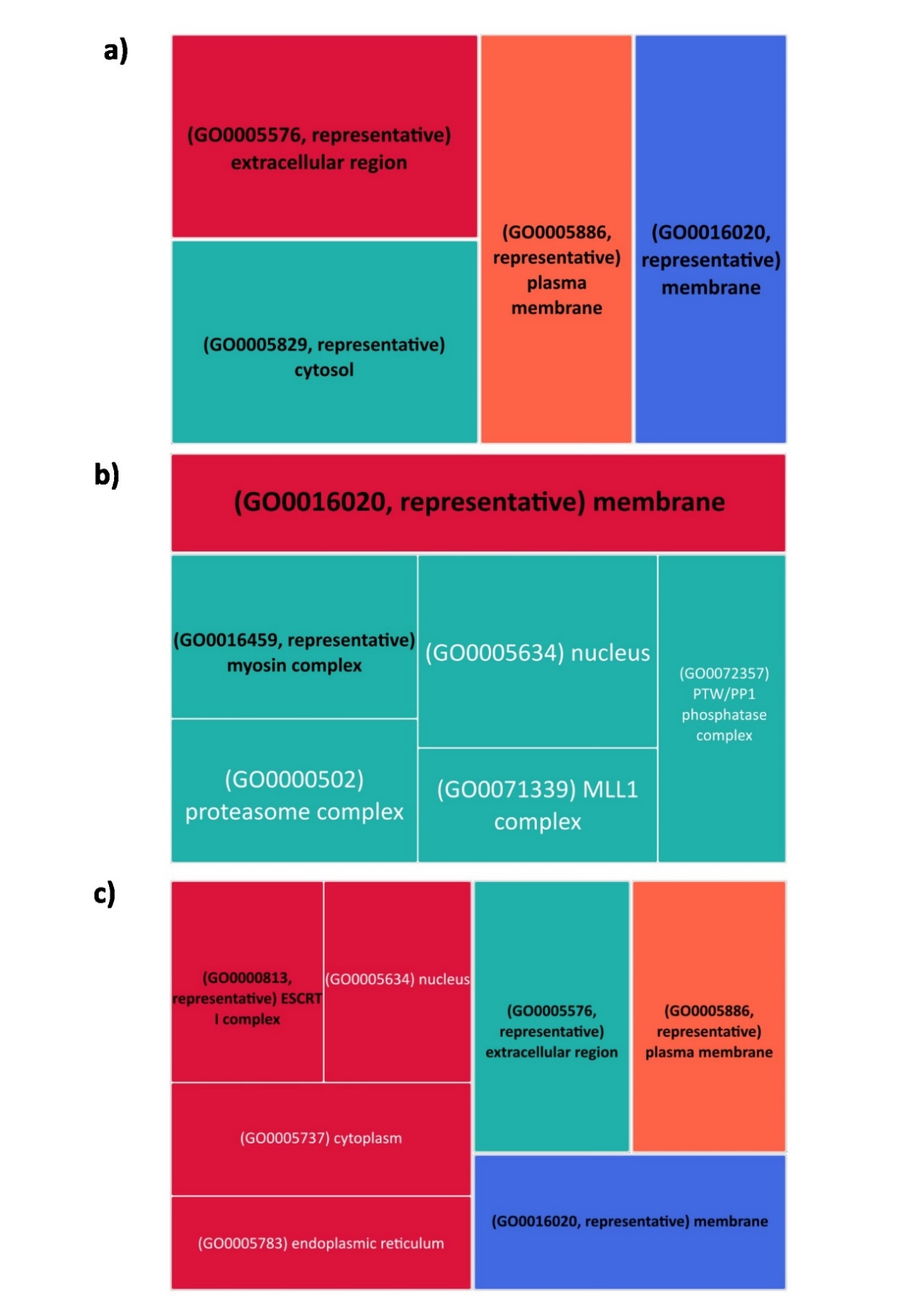


Figure S2. Treemap showing the cellular components in which are involved the genes detected as candidate to be under selection due to the pollution in the comparison: a) PEL-SFM, b) MEL-SFM and, c) IM-SFM. GO terms in black indicate the representative term: terms that are classified as similar after the semantic reduction process. PEL: Pelvin, MEL: Melipilla, IM: Isla de Maipo, SFM: San Francisco de Mostazal.
